# A Case of Puerperal Mania from Suppression of the Lochia; and One of Mania a Potu Complicated with Pleurisy

**Published:** 1846-12

**Authors:** James M. Larkins

**Affiliations:** Charlotte, Tenn.


ART. II.—*A Case of Puerperal Mania from Suppression of the Lochia; and one of Mania a Potu complicated with Pleurisy.* By JAMES M. LARKINS, M.D., of Charlotte, Tenn.

Mrs. C., aged about 28 years, had been in a state of mental derangement for a month when I saw her at the beginning of June. The history of her case was as follows: She had been a delicate girl, her menstrual discharge having been attended with pain and difficulty; she was married at the age of 20 years, and is the mother of three children, the youngest of which was nearly three months old when I was called to visit her. In her last confinement she had been attended by an ignorant midwife, who left her in the hands of a still more ignorant nurse. The nurse fancying there was something amiss in the lochial discharge undertook to suppress it by astringents, in which she was but too successful. The discharge, which ceased soon after delivery, was never reestablished, and the mind of the patient in a short time became excited and finally deranged. Her first medical attendant unfortunately knew little about diagnosis, and mistreated her case. He pronounced the affection one of pure mania, and resorted to the treatment usual in ordinary cases of that malady. Blisters were applied to the nape of her neck, and the cold dash employed.

When I saw the patient she presented the following appearances: Her eyes were blank, and her face expressive of great fear and distress; she was sleepless; her lower extremities cold; body alternately hot and dry, and suffused with perspiration; pressure over the region of the uterus caused her countenance to express pain though she did not complain of it; her language was wild, incoherent, and often obscene; though greatly exhausted, her muscular efforts were violent, so that she was restrained with difficulty. I adopted the following treatment: A large pill of asafœtida was given every hour or two, and a teaspoonful of sulphuric ether every four or five hours; her bowels were kept soluble and occasionally purged by hiera picra; the tepid hip bath was used once or twice daily; and she took every six hours a teaspoonful of a tonic mixture composed of sulphate of quinine and nitric acid.

The first night after this plan was adopted the patient slept quietly, and from that hour began rapidly to improve. In a fortnight she was restored to health of mind and body.

The following case of *mania a potu*, complicated with pleurisy, I had an opportunity of observing in Clinton, Ky., in March last. The patient who was a school teacher by occupation, was in the county jail waiting the session of Court by which the question of lunacy was to be tried. I found him with true pleuritic symptoms, but soon recognised the *mania* as that species induced by *drink.* He was accordingly removed from the jail to a private dwelling. The symptoms were of extreme severity—the tremors such as to shake his bed, the mental disturbance excessive; his tongue red and pointed; cough and pain in the side.

Bleeding was suggested by his medical attendant, but was not adopted on my dissenting from its propriety. A blister was applied over nearly the entire chest; oxymel of squills and mucilaginous drinks were given, and cold water poured over his head. In addition, for the cerebral symptoms, calomel and opium, 2 grains of the latter to 3 of the former, were administered every two hours. At first we tried smaller doses of opium but found them insufficient. In ten days from the adoption of this course the patient was relieved of the pleuritic symptoms, and also of his delirium.

July 15th, 1846.

